# Effects of aging-related muscle degeneration on dynamic stability during walking: a musculoskeletal computer simulation study

**DOI:** 10.3389/fbioe.2024.1524751

**Published:** 2025-01-13

**Authors:** Shoma Kudo, Masahiro Fujimoto, Akinori Nagano

**Affiliations:** ^1^ Health and Medical Research Institute, National Institute of Advanced Industrial Science and Technology (AIST), Kagawa, Japan; ^2^ College of Sport and Health Science, Ritsumeikan University, Shiga, Japan

**Keywords:** gait analysis, balance control, physiological changes, margin of stability (MOS), fall prevention, aging

## Abstract

**Introduction:**

Aging-related deficits in the physiological properties of skeletal muscles limit the control of dynamic stability during walking. However, the specific causal relationships between these factors remain unclear. This study evaluated the effects of aging-related deficits in muscle properties on dynamic stability during walking.

**Methods:**

Walking movements were simulated using two-dimensional musculoskeletal models consisting of 18 Hill-type muscles. To assess the effects of aging-related deficits in muscle function on dynamic stability during walking, five models with different muscle properties were created, namely young adult (YA) and older adult (OA) models, models with reduced maximum isometric muscle force, reduced maximum muscle contraction velocity, and prolonged muscle deactivation time (∆F, ∆V, and ∆T models, respectively). The margin of stability (MoS) was used as a measure of dynamic stability during walking.

**Results and Discussion:**

The MoS value of the OA model was greater than that of the YA model, and the ∆F model yielded a larger MoS value than those of the ∆V and ∆T models. Therefore, the OA model achieved a more dynamically stable state than the YA model and the ∆F model required a more stable state to sustain continuous walking compared to the ∆V and ∆T models. These findings indicate that aging-related deficits in muscle function limit the control of dynamic stability during walking with the degeneration of maximum isometric muscle force being the most influential factor. These findings could aid in the development of an intervention program to reduce the risk of falls in older adults effectively.

## 1 Introduction

Falling is the leading cause of serious injuries in older adults (Tinetti et al., 1988) and their risk increases with age. The alteration in risk of falling is thought to be linked to aging-related deficits in the physiological properties of skeletal muscles (Landi et al., 2012; Yamada et al., 2013; Yamaguchi and Masani, 2022). However, the precise impact and extent of muscle property deficits on stability during walking remain unclear. Therefore, achieving a better understanding of how changes in muscle properties affect walking stability is essential for enhancing the implementation of fall-prevention interventions.

Although several experimental studies have documented that the physiological properties of skeletal muscles and stability during walking decline with age, the causal relationships between these factors have not been revealed. Compared to young adults, healthy older adults exhibit prolonged muscle deactivation time (Doherty et al., 1993), as well as loss of muscle strength (Brooks and Faulkner, 1994; Delmonico et al., 2009) and a reduced rate of force development (Alcazar et al., 2020). Such degeneration of muscle properties is thought to compel older adults to adopt a more conservative strategy than young adults to ensure stability during walking (Fujimoto and Chou, 2016). However, physiological changes in skeletal muscles cannot be independently intervened in human experiments. This limitation makes it difficult to determine the causal relationship between a particular physiological change and dynamic stability during walking.

Compared with experimental studies, computational forward dynamics simulations using a musculoskeletal model provide a more practical alternative approach. Physiological changes in skeletal muscles can be independently controlled in neuromechanical models of human movement and this capability enables exploring how skeletal muscle properties influence body motion. This advantage allows assessing effects of musculoskeletal changes on overall motion (Song and Geyer, 2018; Arakawa et al., 2021; Schreff et al., 2022), investigating the impact of injuries on physical activity (Donno et al., 2022), and analyzing body movement improvements post-surgery (Tzanetis et al., 2021). Therefore, predictive neuromechanical simulations can provide evidence that physiological changes in skeletal muscles influence dynamic stability during walking.

In this study, we evaluated the effects of aging-related deficits in muscle properties on dynamic stability during walking. Walking movements were simulated using two-dimensional musculoskeletal models consisting of 18 Hill-type muscles. Muscle deactivation time, maximum isometric muscle force, and maximum muscle contraction velocity were adjusted to assess the effect of aging-related deficits in muscle function on dynamic stability during walking.

## 2 Methods

Walking motion was simulated using five different forward dynamics models of the human musculoskeletal system, which differed in their muscle properties, namely, young adults (YA) and older adults (OA) models; additionally, models with prolonged muscle deactivation time, reduced maximum isometric muscle force, and reduced maximum muscle contraction velocity (∆T, ∆F, and ∆V models, respectively). The OA model incorporated all the adjustments of the ∆T, ∆F, and ∆V models. These adjustments reflected changes typically seen from ages 30 to 70, as described by Thelen (2003). Numerical optimization techniques were used to derive a set of control parameters that produced human-like walking in the model. The dynamic stability (Hof et al., 2005) and spatiotemporal parameters of walking simulated by the YA and OA models were quantified to investigate whether aging-related deficits in muscle function affect dynamic stability during walking. The values derived from the ∆T, ∆F, and ∆V models were also evaluated to reveal which aging-related deficits in muscle function have the greatest effect on the stability of walking.

### 2.1 Overview of musculoskeletal computer simulation system

**FIGURE 1:**
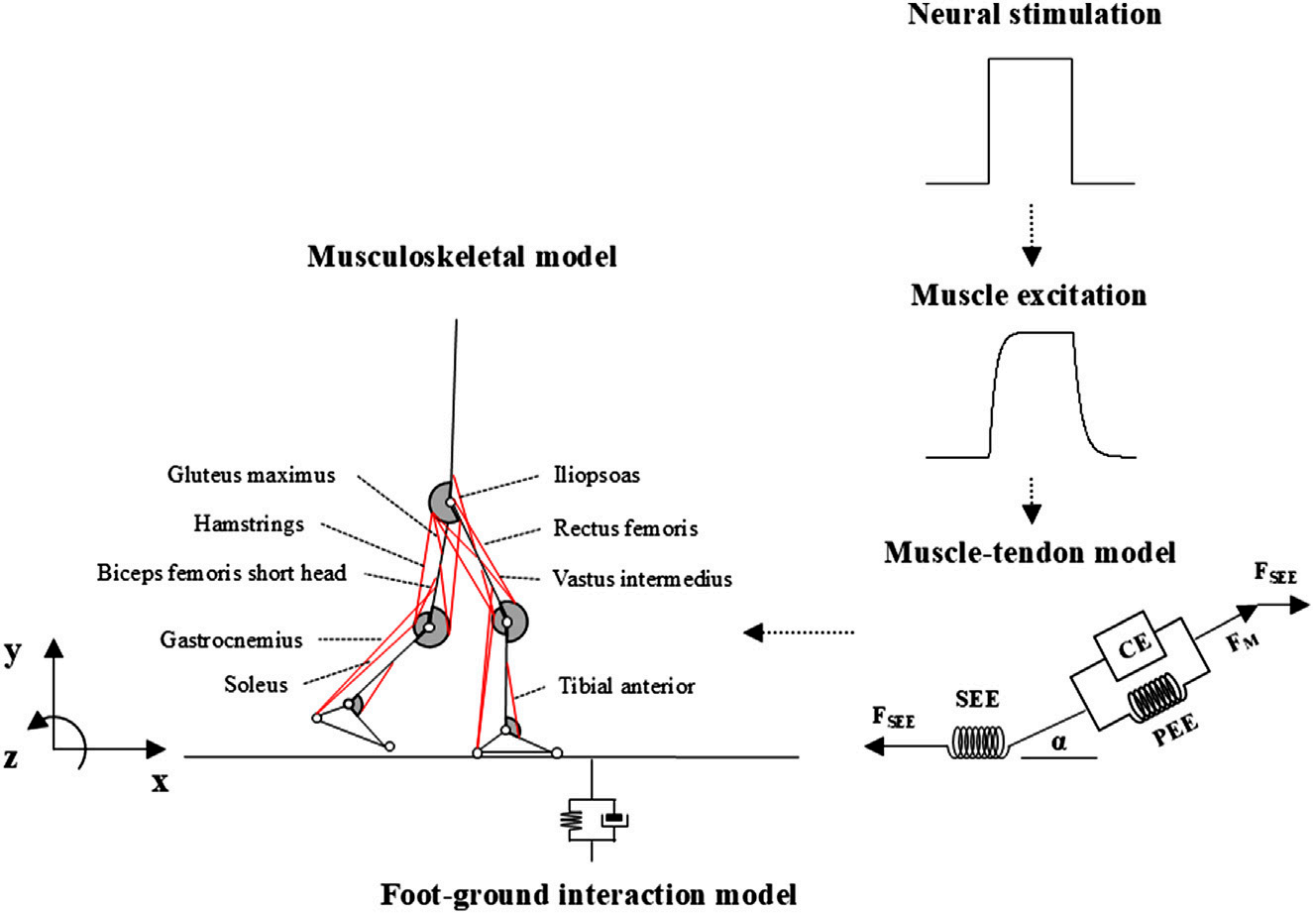
Overview of the musculoskeletal computer simulation system.

The musculoskeletal computer simulation system used in this study included a skeletal, muscular, and external environment interaction model (i.e., foot/ground interaction, see Figure 1). The inputs for the system were time-independent neural stimulation profiles for each muscle that passed through a first-order process, resulting in the activation of the contractile elements (CEs) in the Hill-type muscle model. The musculoskeletal model was developed based on the forces generated by the series elastic element (SEE), ground reaction forces (GRFs), and passive moments of each joint ( $M_{pass}$ ). The musculoskeletal computer simulation system was implemented using the commercial package Motion Genesis (Motion Genesis LLC, Stanford, CA, United States), which is linked to C-language code.

### 2.2 Musculoskeletal model

The skeleton of the human body was modeled using seven rigid-body segments representing the head-arms-trunk (HAT), right and left upper and lower legs, and feet (Figure 1). The complete model had 10 degrees of freedom (DOFs), the HAT had four DOFs (three translations and one rotation about the mediolateral axis of the global reference frame), and each hip, knee, and ankle joint had one DOF (flexion/extension and dorsiflexion/plantar flexion). The body height and mass of the model were set to 1.74 m and 72.80 kg, respectively. The anthropological parameter values of the model (length, mass, position of the mass center, and moment of inertia of segments) were derived from a previous study (de Leva, 1996). The passive joint moments that exist in the lower-limb joints (Riener and Edrich, 1999) were also implemented in our model. Foot-ground interaction was simulated with two contact elements placed at the heel and toe for each foot, generating vertical forces as damped quadratic springs and shear forces approximating the Coulomb friction (Miller and Hamill, 2015).

Eighteen Hill-type muscle models representing the major muscles in the human lower extremities were implemented in the skeletal model, namely, the m. iliopsoas (ILIA), m. glutei (GMAXI), hamstrings (HAM), m. rectus femoris (RECTF), mm. vasti (VASTI), m. biceps femoris short head (BFES), m. gastrocnemius (GAMS), m. soleus (SOLEU), and m. tibialis anterior (TIBAN) in each leg (Figure 1). The paths of these muscles were determined based on the coordinates of the origin, via point, and insertion of the muscles reported by Delp (1990).

The force-producing properties of the muscle were defined by five parameters: maximum isometric force, optimal fiber length, pennation angle, tendon slack length, and maximum contraction velocity, along with four curves: tendon force-length, active and passive muscle force-length, and muscle force-velocity. The active force-length curve scaled with muscle activation from 0 (no activation) to 1 (full activation). These four relationships were modeled with nonlinear functions (van Soest and Bobbert, 1993; Nagano and Gerritsen, 2001; Umberger et al., 2003). The value for the muscle parameters in the YA model was determined by referencing previous reports. The maximum isometric muscle force of the YA model was calculated by multiplying specific tension (60 N/cm^2^, Rajagopal et al., 2016) by the physiological cross-sectional area (PCSA). PCSA data, originally from a 1.83 m, 91 kg, 37-year-old specimen (Friederich and Brand, 1990), were scaled to 1.741 m, 73 kg for the YA model. The maximum contraction velocity of each muscle in the YA model was taken to be 12 optimal muscle fiber lengths per second (Umberger et al., 2003). Values for the optimal fiber length and pennation angle were determined based on Delp (1990).

The muscle activation dynamics were modeled with an ordinary differential equation (He et al., 1991). This equation captures the delay between neural stimulation of the muscle and its transition to an active state. The activation and deactivation times in the YA model were set at 55 and 65 ms, respectively (Nagano and Gerritsen, 2001).

### 2.3 Adjustment of muscle parameters for elderly muscle function

To simulate age-related declines in muscle function, adjustments were made to the values of deactivation time, maximum isometric force, and maximum contraction velocity in the OA, ∆T, ∆F, and ∆V models, reflecting the muscle atrophy and remodeling typically observed between the ages of approximately 30 and 70. The specific amount of degeneration was referred to the findings of Thelen (2003). In the OA model, compared to the YA model, muscle deactivation time was extended by 20%, maximum isometric muscle force was reduced by 30%, and maximum muscle contraction velocity was reduced by 20%. Each of the adjustments for muscle deactivation time, maximum isometric muscle force, and maximum muscle contraction velocity were applied independently in the ∆T, ∆F, and ∆V models, respectively. These functional declines are thought to result from a reduction in the size and number of muscle fibers with aging (Lexell et al., 1988; Nilwik et al., 2013), as well as a decreased rate of calcium ion uptake by the sarcoplasmic reticulum (Larsson and Salviati, 1989).

### 2.4 Optimization and control

**FIGURE 2:**
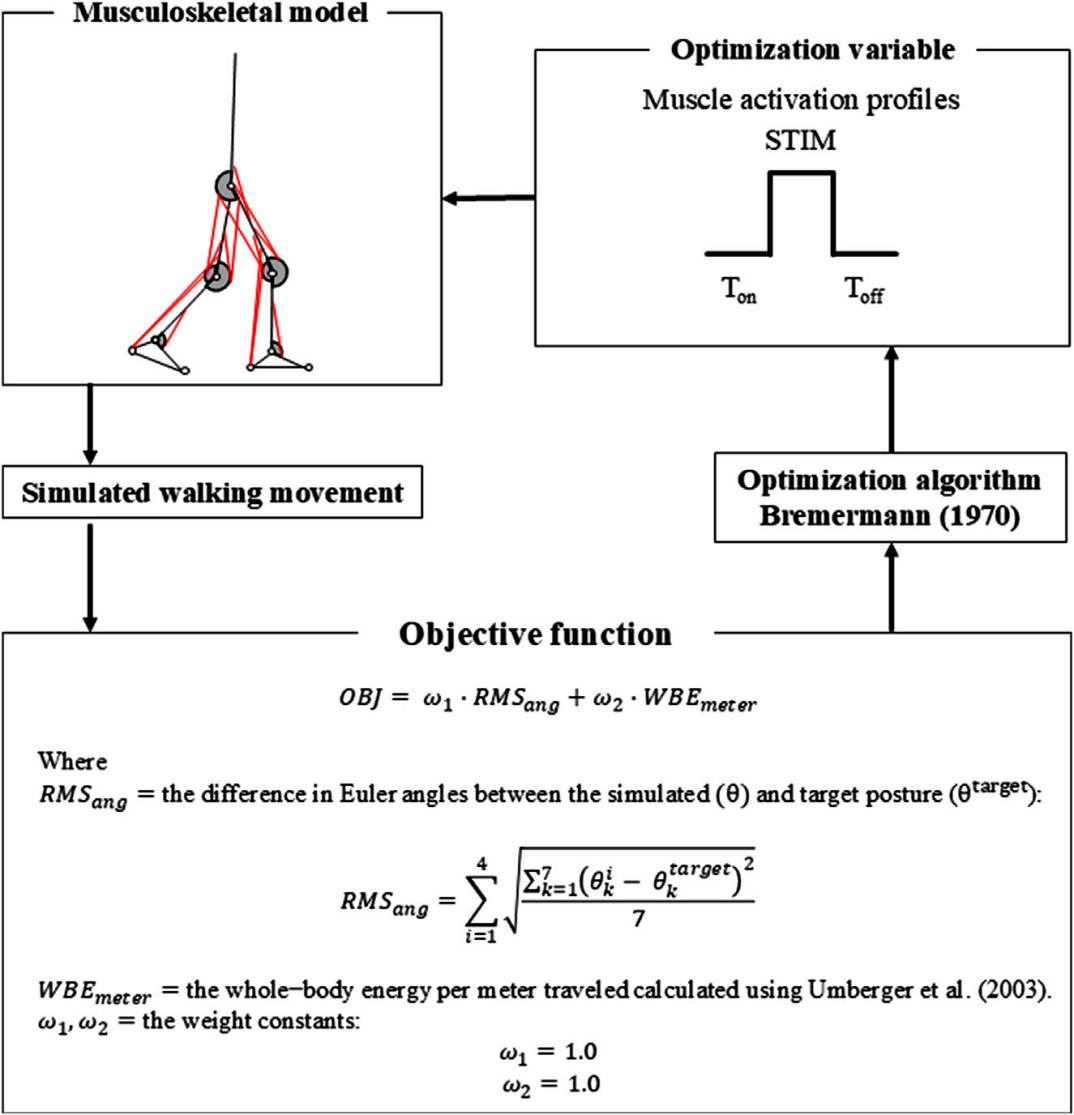
Flowchart describing details of the numerical optimization process.

Numerical optimization was performed to derive muscle activation patterns that yielded walking with minimum energy expenditure. The inputs for the neuromusculoskeletal model, used to simulate walking motion, comprised 18 muscle activation profiles (Figure 2), each specified by three values: onset time, offset time, and amplitude of stimulation. The muscle stimulation profiles in the model’s left leg were assumed to be identical to, but 50% out of phase, with those in the right leg (Umberger, 2010), in order to reduce the number of variables used in the optimization process.

Optimal muscle stimulation profiles for generating walking motions were derived through numerical optimization. Optimizations were performed using a modified version of an algorithm proposed in a previous study (Bremermann, 1970), which was implemented in MATLAB (MathWorks Inc., Natick, MA, United States). The objective function was defined based on a previous study that successfully simulated a human walking motion (Nagano et al., 2005). The goal of numerical optimization was to minimize the following objective function consisting of two separate terms:

$$Objective\;function=\omega_{1}\cdot {RMS}_{ang}+\omega_{2}\cdot {WBE}_{meter}$$

where $\omega_{1}$ and $\omega_{2}$ represent the weight constants ( $\omega_{1}=\omega_{2}=1.0$ ). The ${RMS}_{ang}$ indicates the sum of difference in Euler angles of each body segment between the simulated posture at the end of step and a typical posture at the instant of toe-off during walking (one Euler angle for 7 segments = 7 angles):

$${RMS}_{ang}=\sum_{i=1}^{4}\sqrt{\frac{\sum_{k=1}^{7}{\left(\theta_{k}^{i}-\theta_{k}^{target}\right)}^{2}}{7}},$$

where θ represents the angle of the *k*-th segment at the toe-off during the *i*-th step and $\theta^{target}$ represents the target posture angle. The target posture in the instance of the toe-off was preliminarily obtained from the experimental data. The ${WBE}_{meter}$ represents the whole-body energy expenditure per meter traveled:

$${WBE}_{meter}=\frac{\int_{0}^{t_{f}}{\dot{E}}_{total}^{M}\;}{X_{CoM}\left(t_{f}\right)-X_{CoM}\left(0\right)},$$

where ${\dot{E}}_{total}^{M}$ is the rate of metabolic energy consumption in the model, and $X_{CoM}\left(0\right)$ and $X_{CoM}\left(t_{f}\right)$ are the initial and final horizontal positions of the whole-body center of mass, respectively. Details of the muscle energy consumption model were given by Umberger et al. (2003).

Four steps of the walking motion, defined as the period between two successive foot contacts of the right and left limbs, were simulated. The simulation duration was defined based on the spatiotemporal characteristics preliminarily observed in experimental data. The simulation duration was set to 2.24 s, assuming the duration of one step was 0.56 s.

### 2.5 Analysis

The kinematic data from the model during the second and third steps were used for analysis. The toe-off times were determined based on the vertical component of the ground reaction force (threshold of 0.05% of body weight).

The margin of stability (MoS) was calculated as a measure of dynamic stability during walking (Hof et al., 2005) and was compared between models. The MoS is defined as the shortest distance in the anteroposterior direction between the velocity-adjusted position of the center of mass (XCoM) and the boundary of base of support (BoS) at the time of left toe-off in the second step. The toe on the right foot was used as the boundary of the BoS. The MoS was calculated as follows:

$$D_{XCoM}=BoS-XCoM$$

where

$$XCoM=P_{CoM}+\frac{V_{CoM}}{\sqrt{\frac{g}{l}}}$$

where $P_{CoM}$ , $V_{CoM}$ , g , and l are the position and velocity of the center of mass at the time of toe-off, gravitational acceleration, and Euclidian distance from the ankle to the $P_{CoM}$ , respectively. When the XCoM is inside the BoS (i.e., MoS is positive), the model is in a dynamically stable state. However, when the XCoM is outside the BoS (i.e., the MoS is negative), the model is dynamically unstable.

The step length and $V_{CoM}$ were used as spatiotemporal parameters to evaluate how the model controlled dynamic stability during walking because these parameters affect the boundary of the BoS and the position of the XCoM, respectively. The step length was calculated as the distance between the right and left toes at the time of toe-off.

The validity of simulated walking motions was evaluated through the cross-correlation analysis with joint kinematics data from empirical walking trials. The empirical dataset was sourced from the publicly accessible gait database (Kobayashi et al., 2019). The dataset used for the validation analysis included measurements of flexion-extension angles of the lower limb joints from a sample of 20 individuals, comprising equal numbers of males and females, divided into two age categories: the 30s (YA_empirical_) and the 70s (OA_empirical_). The study assessed the simulated data by analyzing the cross-correlations between the kinematic data from the YA model and the YA_empirical_, as well as between the OA model and the OA_empirical_. Additionally, the averaged value of MoS, step length and $V_{CoM}$ for both the YA_empirical_ and OA_empirical_ were also calculated to serve as reference data.

## 3 Results

**FIGURE 3:**
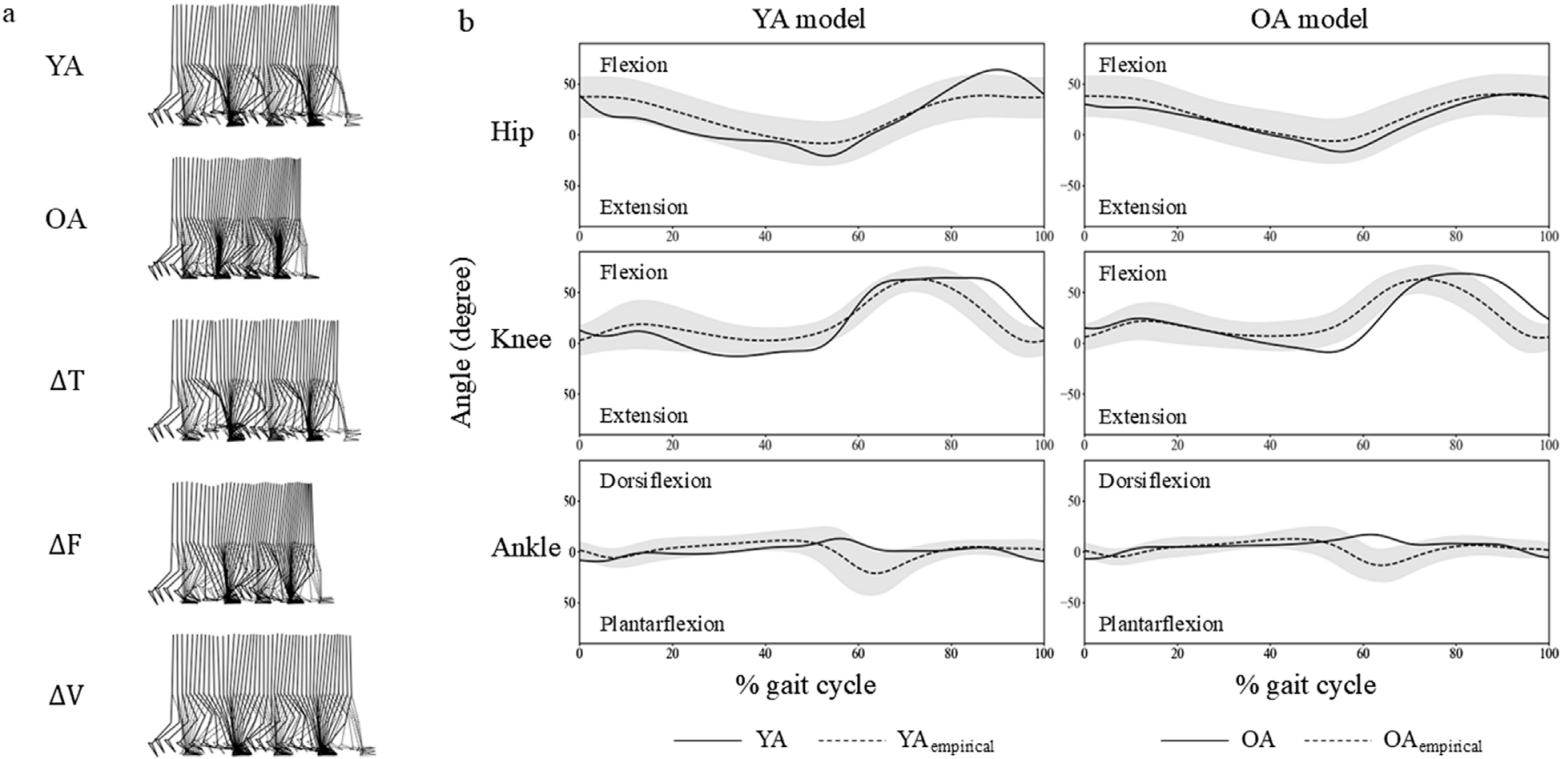
**(A)** Snapshots of four-step walking sequences from the musculoskeletal models used in this study. **(B)** Hip, knee, and ankle joint angles from the YA and OA models are shown with solid lines. Shaded areas represent three standard deviations from the mean based on a sample of 20 individuals, marked with dashed lines. These individuals are categorized into two age groups: the 30s (YA_empirical_) and the 70s (OA_empirical_).

The study successfully generated a four-step walking motion across all established models (Figure 3). The average walking velocities from the second and third steps were 1.26 m/s, 0.98 m/s, 1.27 m/s, 1.08 m/s, 1.36 m/s, for YA, OA, ∆T, ∆F, and ∆V models, respectively. The averaged cross-correlation values between the YA model and the 30s age group were 0.71 overall, with individual values of 0.85, 0.91, and 0.37 for the hip, knee, and ankle joint angles, respectively. Similarly, the OA model and the 70s age group demonstrated an overall cross-correlation of 0.79, with individual values of 0.98, 0.92, and 0.48 for the hip, knee, and ankle joint angles, respectively.

**FIGURE 4:**
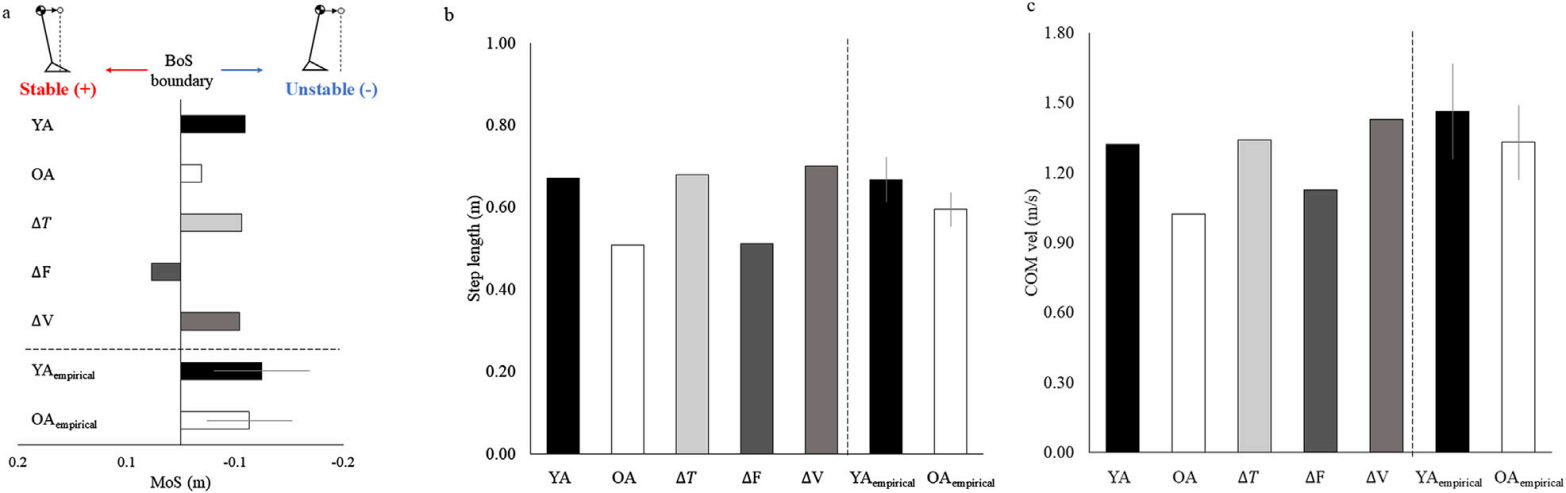
**(A)** MoS, **(B)** step length, and **(C)** walking velocity at the time of toe-off for each model.

The MoS value were −0.06 m, −0.02 m, −0.06 m, 0.03 m, and −0.05 m, for the YA, OA, ∆T, ∆F, and ∆V models, respectively. The MoS value of the OA model was higher than that of the YA model (Figure 4A). When comparing the values of the MoS between the ∆T, ∆F, and ∆V models, the value of the ∆F model was larger than those of the ∆T and ∆V models. The average MoS value of the YA_empirical_ and OA_empirical_ were −0.06 m and −0.08 m, respectively. Consistent with the simulated data, the OA_empirical_ displayed a higher MoS value than the YA_empirical_.

The step length were 0.67 m, 0.51 m, 0.68 m, 0.51 m, and 0.70 m, for the YA, OA, ∆T, ∆F, and ∆V models, respectively. The step length of the YA model was larger than that of the OA model (Figure 4B). When comparing the values of the step length between the ∆T, ∆F, and ∆V models, the value of the ∆F model was smaller than those of the ∆T and ∆V models. The average step length of the YA_empirical_ and OA_empirical_ were 0.67 m and 0.06 m, respectively. Consistent with the simulated data, the YA_empirical_ displayed a higher step length than the OA_empirical_.

The $V_{CoM}$ value were 1.32 m/s, 1.02 m/s, 1.34 m/s, 1.13 m/s, and 1.43 m/s, for the YA, OA, ∆T, ∆F, and ∆V models, respectively. The $V_{CoM}$ value of the YA model was higher than that of the OA model (Figure 4C). When comparing the values between the ∆T, ∆F, and ∆V models, the value of the ∆F model was smaller than those of the ∆T and ∆V models. The average $V_{CoM}$ value of the YA_empirical_ and OA_empirical_ were 1.46 m/s and 1.33 m/s, respectively. Consistent with the simulated data, the YA_empirical_ displayed a higher $V_{CoM}$ value than the OA_empirical_.

**FIGURE 5:**
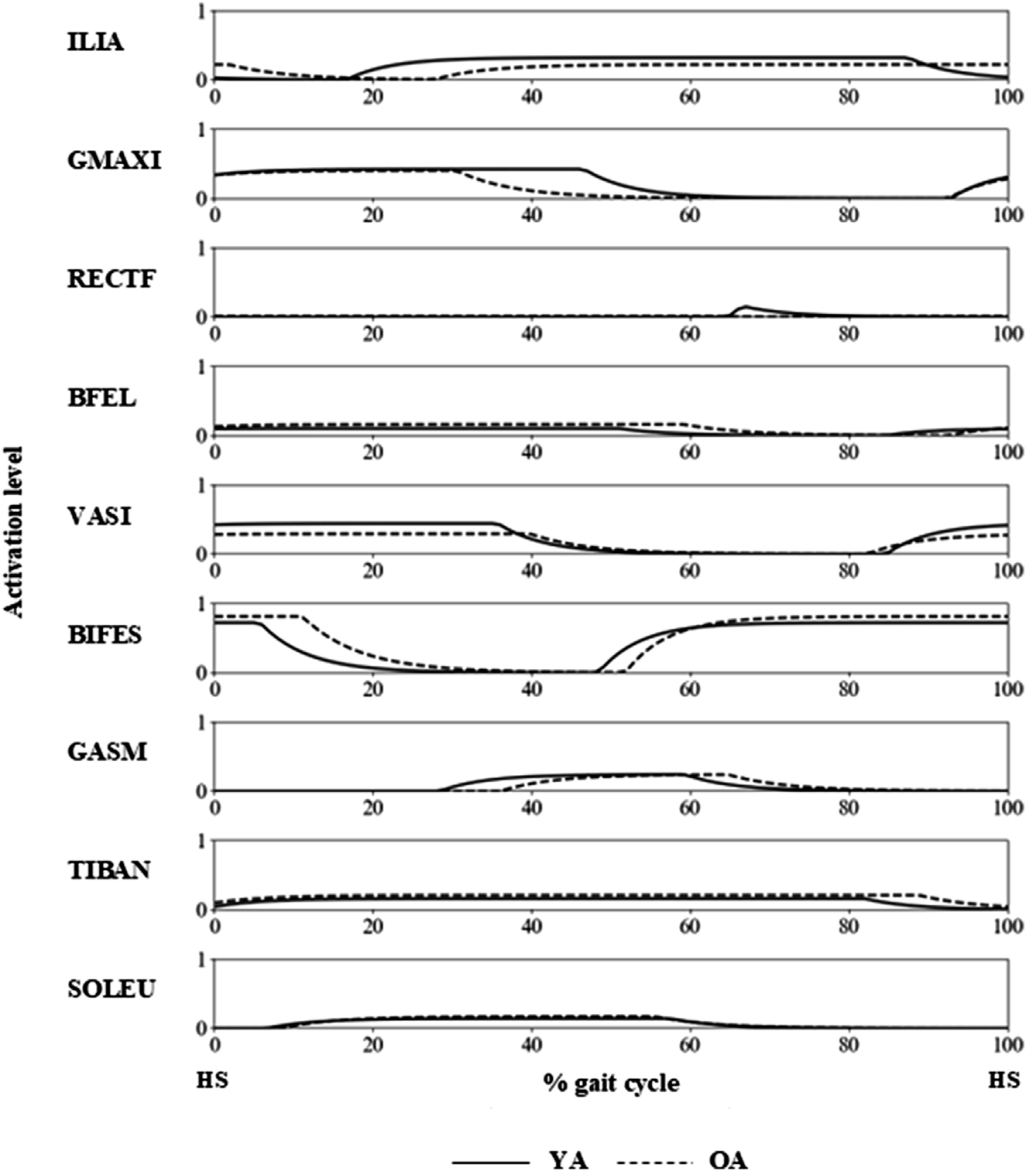
Muscle activation profiles of the YA and OA models during a gait cycle, with HS indicating the timing of the right heel strike.

The joint angle profiles were qualitatively similar between the YA and OA models throughout the gait cycle (Figure 3B). However, a subtle difference was observed in the ankle joint around 60% of the gait cycle, approximately corresponding to the toe-off phase. At this point, the ankle in the OA model exhibited greater dorsiflexion compared to the YA model. Regarding the muscle activation profiles, the activity levels were similar across all muscles in both the YA and OA models (Figure 5). However, slight differences were noted in the timing of muscle activation onset and offset.

## 4 Discussion

We investigated the effects of aging-related deficits in muscle properties on dynamic stability during walking. Four continuous walking steps were simulated using five musculoskeletal models with different muscle functions. The MoS at the toe-off of the second step was used as a measure of dynamic stability during walking. The MoS value of the OA model was higher than that of the YA model, indicating that the OA model had to walk in a more dynamically stable state than the YA model. The ∆F model also exhibited larger MoS values than the ∆V and ∆T models, indicating that the ∆F model required a more stable state to sustain continuous walking compared to the ∆V and ∆T models. These findings suggest that aging-related deficits in muscle function limit the control of dynamic stability during walking and that the degeneration of maximum isometric muscle force is the most influential factor affecting dynamic stability during walking.

The larger MoS value in the OA model compared to that of the YA model indicated that the OA model walked more dynamically stable than the YA model. This finding implies that aging-related deficits in muscle function forced the model to walk in a dynamically stable state to execute continuous walking. This result is consistent with a previous experimental study, which demonstrated that older adults tend to walk under a more conservative strategy than healthy young adults, in which the whole-body CoM is positioned close to the BoS at the instance of toe-off (Fujimoto and Chou, 2016). The current study demonstrated the pure effect of aging-related deficits in muscle function on dynamic stability during walking and suggested that deficits in muscle function limit the control of dynamic stability during walking. These findings suggest the importance of maintaining muscle function during aging to control dynamic stability during walking.

The ∆F model walked in a more dynamically stable state compared to the ∆V and ∆T models, as evidenced by its larger MoS value. The ∆F model also exhibited a shorter step length and smaller CoM velocity compared to the ∆V and ∆T models. These reductions in step length and CoM velocity appeared to stabilize the CoM motion state, resulting in an increase in dynamic stability during walking. These findings suggest that an aging-related deficit in maximum isometric muscle force is the most important factor for maintaining dynamic stability during walking, rather than the extension of muscle deactivation time or slowing of maximum muscle contraction velocity.

The models with individual deterioration in muscle functions (ΔT, ΔF, and ΔV models) highlighted the impact on dynamic stability for each function, whereas the model with simultaneous degeneration of all muscle functions (OA model) demonstrated the averaged effects of these impairments. The differences in MoS between the YA model and the other models were −0.040 m, 0.003 m, −0.086 m, and −0.005 m for the OA, ΔT, ΔF, and ΔV models, respectively. The average difference in the MoS for the models with individual impairments in muscle function was −0.031 m, which is comparable to the difference observed in the OA model. This implies that the influence on dynamic stability from the deterioration in each muscle function would not accumulate monotonously. Therefore, these findings suggest that the effect of aging-related deficits in individual muscle functions would not independently affect the dynamic stability during walking.

This study has several limitations that must be acknowledged. The musculoskeletal model employed was a simplified two-dimensional representation with a limited number of muscle models, and it excluded neural and sensory system models. Falls among the elderly frequently occur in multiple directions (Robinovitch et al., 2013), and are exacerbated by aging, which affects postural stability due to changes in muscular, neural, and sensory functions (van der Kruk et al., 2021). This indicates the need for a more advanced simulation system with a three-dimensional skeletal model integrating muscle, neural, and sensory systems for a more accurate assessment of stability. It should also be noted that employing additional condition could be beneficial to assess dynamic stability in walking. For instance, simulating walking over uneven terrain (Haeufle et al., 2018; Schreff et al., 2022) or assessing the system’s response to external perturbations (Murai and Yamane, 2011) could provide a more comprehensive evaluation of the simulation system’s robustness. Another limitation was assuming consistent step timing and symmetrical muscle activation profiles for both legs, potentially missing gait cadence variations. However, the model’s validity is underscored by the cross-correlation values between the simulation model and empirical data, which exceeded 0.7—a threshold typically regarded as indicating a strong correlation—for both the YA and OA models. These findings suggest that the model used in this study reflects the effect of age-related muscle deficits on dynamic stability during walking.

In conclusion, this study confirmed that aging-related degeneration of muscle properties impairs dynamic stability during walking according to the results of forward dynamics simulations. Additionally, we determined that the degeneration of maximum isometric muscle force is a more significant limiting factor for controlling dynamic stability during walking compared to the maximum muscle contraction velocity and deactivation time. These findings could aid in the development of an intervention program to reduce the risk of falls in older adults effectively.

## Data Availability

The original contributions presented in the study are included in the article/supplementary material, further inquiries can be directed to the corresponding author.
